# Decreasing hydrophobicity or shielding hydrophobic areas of CH2 attenuates low pH-induced IgG4 aggregation

**DOI:** 10.3389/fbioe.2023.1257665

**Published:** 2023-08-29

**Authors:** Qiang Wu, Chunlai Cao, Suzhen Wei, Hua He, Kangyue Chen, Lijuan Su, Qiulian Liu, Shuang Li, Yongjie Lai, Jing Li

**Affiliations:** ^1^ School of Biology and Biological Engineering, South China University of Technology, Guangzhou, China; ^2^ Zhuhai United Laboratories Co., Ltd., Zhuhai, Guangdong, China; ^3^ The United Biotechnology (Zhuhai Hengqin) Co., Ltd., Zhuhai, Guangdong, China; ^4^ Department of Microbiology and Immunology, Zunyi Medical University (Zhuhai Campus), Zhuhai, Guangdong, China

**Keywords:** aggregation, CH2, Fc, hydrophobicity, mannose, protein A chromatography, sucrose, viral inactivation

## Abstract

Protein aggregation is a major challenge in the development of therapeutic monoclonal antibodies (mAbs). Several stressors can cause protein aggregation, including temperature shifts, mechanical forces, freezing-thawing cycles, oxidants, reductants, and extreme pH. When antibodies are exposed to low pH conditions, aggregation increases dramatically. However, low pH treatment is widely used in protein A affinity chromatography and low pH viral inactivation procedures. In the development of an IgG4 subclass antibody, mAb1-IgG4 showed a strong tendency to aggregate when temporarily exposed to low pH conditions. Our findings showed that the aggregation of mAb1-IgG4 under low pH conditions is determined by the stability of the Fc. The CH2 domain is the least stable domain in mAb1-IgG4. The L309E, Q311D, and Q311E mutations in the CH2 domain significantly reduced the aggregation propensity, which could be attributed to a reduction in the hydrophobicity of the CH2 domain. Protein stabilizers, such as sucrose and mannose, could also attenuate low pH-induced mAb1-IgG4 aggregation by shielding hydrophobic areas and increasing protein stability. Our findings provide valuable strategies for managing the aggregation of protein therapeutics with a human IgG4 backbone.

## Introduction

Antibodies are biologically active proteins produced by immune cells to defend against invading pathogens. Monoclonal antibodies (mAbs) are antibodies derived from identical B cells. Due to their high selectivity, potency, and multiple biological functions, mAbs have been one of the fastest-growing classes of therapeutics. To date, more than 100 monoclonal antibodies have been approved by the FDA for the treatment of cancer, chronic diseases, and autoimmune disorders (Mullard, 2021). Human IgG consists of four subclasses referred to as IgG1, IgG2, IgG3, and IgG4. IgG1 and IgG3 are much more potent than IgG2 and IgG4 in triggering effector functions such as antibody-dependent cell-mediated cytotoxicity (ADCC) and complement-dependent cytotoxicity (CDC) (Schroeder and Cavacini, 2010; Jiang et al., 2011). Due to its shorter half-life, IgG3 has not yet been exploited as a therapeutic antibody. On the other hand, IgG2 and IgG4 backbones have been specially selected when only blocking function is required, especially in autoimmune disorders (Beers et al., 2016).

The biological activity of proteins is closely related to their conformational structure and stability. Aggregation is a common and disturbing manifestation of protein instability (Wang, 2005). Protein aggregates are formed by the association of monomers, resulting in higher molecular weight oligomers. They can be classified as soluble or insoluble, covalent or non-covalent, reversible or non-reversible aggregates (Wang and Roberts, 2018; Ebo et al., 2020). Elucidation of the underlying mechanism of aggregation may provide feasible approaches to prevent protein aggregation (Wang, 2005). Many factors contribute to protein aggregation, which can be classified as structural (internal) or environmental (external). It has been reported that the primary amino acid sequence plays a key role in determining a protein’s conformational structure, surface charge distribution, hydrophobicity, and finally the propensity to aggregate (Anfinsen, 1973; Marks et al., 2012; Liu et al., 2021; Zhou et al., 2022; Housmans et al., 2023). The aggregation propensity of antibodies could be determined by variable domains (VH and VL), CH2 domain, or CH3 domain (Andersen et al., 2010; Brummitt et al., 2011; Dudgeon et al., 2012; Latypov et al., 2012; Iacob et al., 2013; Zhang-van Enk et al., 2013; Wu et al., 2014; Sakurai et al., 2015; Majumder et al., 2018; Namisaki et al., 2020). Protein aggregation could also be induced by a variety of external or environmental factors, including temperature shifts, extreme pH, ionic strength, shaking, shearing, and the freezing-thawing cycle. Any factors, either internal or external, that cause complete or partial denaturation of proteins, exposure of hydrophobic patches, or changes in surface charge distribution, could enhance the attraction between protein molecules and accelerate the formation of aggregates.

Multiple studies have reported significant aggregation formation when antibodies were temporarily exposed to low pH conditions (pH 3–4) (Chen et al., 2010; Skamris et al., 2016). However, low pH buffers are used in the protein A affinity chromatography elution step and viral inactivation procedure in monoclonal antibody production (Hober et al., 2007; Bolton et al., 2015; Mazzer et al., 2015; Klutz et al., 2016). During the development of an IgG4 subclass monoclonal antibody, we found that mAb1-IgG4 significantly aggregated under low pH conditions (citrate buffer, pH 3.5 and below). We speculated that the hydrophobic patches on the mAb1-IgG4 predominate in the aggregation under low pH conditions and found out that the CH2 domain plays a key role in mAb1-IgG4 aggregation. In order to improve the stability of mAb1-IgG4 and reduce the protein aggregation in low pH solutions, we performed point mutations to reduce the hydrophobicity of CH2 and supplemented sucrose to shield the hydrophobic patches on CH2 domain, both these two strategies greatly improved the protein stability and reduced the protein aggregation propensity.

## Materials and methods

### Materials

All the mAbs used in this study were produced at Zhuhai United Laboratories Co., Ltd. (Zhuhai, Guangdong province, China), including mAb1-IgG4, mAb1-IgG1, mAb1-IgG2, mAb1-IgG4-L309E, mAb1-IgG4-L309T, mAb1-IgG4-L309S, mAb1-IgG4-Q311D and mAb1-IgG4-Q311E. Each numeral indicated EU numbering. All chemical reagents were purchased from Sigma Aldrich (St. Louis, MO, United States). The IdeS protease was purchased from Promega (Madison, WI, United States). Antibody fragments, F (ab’)_2_ and Fc, were generated by IdeS digestion.

### Low pH incubation

Full-length mAbs and antibody fragments were diluted to a final concentration of 1 mg/mL in 25 mM citrate buffer at different pHs for 1 or 2 h at room temperature. The low pH-treated samples were then neutralized to pH 7.0 with a 2 M Tris-HCl solution (pH 8.5) for further analysis.

### Size exclusion chromatography (SEC)

For intact antibodies, the SEC was performed on an Agilent 1,260 series HPLC system using a TSK-Gel G3000SWXL (7.8 mm × 300 mm, 5.8 μm). Protein samples were diluted to 0.5 mg/mL, and 50 μL of samples were used for analysis. The column was equilibrated with the buffer (10 mM disodium hydrogen phosphate, 10 mM sodium dihydrogen phosphate, and 300 mM sodium chloride, pH 7.0), and isocratic elution was used for chromatographic separation. The flow rate was 0.5 mL/min, and the column temperature was 25°C. The data were collected at 280 nm.

For the antibody fragments, SEC was performed on a Waters 1,260 series UPLC system using a BEH200 SEC column (1.7 mm, 4.6 mm × 150 mm, 200 Å). Protein samples were diluted to 0.5 mg/mL, and 10 μL of samples were used for analysis. The column was equilibrated with the buffer (10 mM disodium hydrogen phosphate, 10 mM sodium dihydrogen phosphate, and 300 mM sodium chloride, pH 7.0), and isocratic elution was used for chromatographic separation. The flow rate was 0.1 mL/min, and the column temperature was 30°C. The data were collected at 280 nm.

### Cation-exchange chromatography (CEX)

The CEX was performed on an Agilent 1,260 series HPLC system with a BioPro IEX SF analytical column (100 × 4.6 mm; 5 μm, YMC Co., Ltd., Kyoto, Japan). Protein samples were diluted to 0.5 mg/mL, and 50 μL of samples were used for analysis. The column was equilibrated with buffer A (30 mM MES, pH 6.2), and the proteins were then eluted with a linear gradient of buffer B (30 mM MES, 500 mM sodium chloride, pH 6.2) from 0% to 30% for 60 min. The flow rate was 0.4 mL/min. The data were collected at 280 nm.

### Hydrophobic interaction chromatography (HIC)

The HIC was performed on an Agilent 1,260 series HPLC system equipped with a MabPac HIC-10 column (4.6 × 100 mm, 5 μm, Thermo Scientific, United States). Protein samples were diluted to 0.5 mg/mL, and 20 μL of samples were used for analysis. The column was equilibrated with buffer A (2 M ammonium sulfate with 0.1 M sodium phosphate, pH 7.0), and the proteins were then eluted with a linear gradient of buffer B (0.1 M sodium phosphate, pH 7.0) from 50% to 100% for 60 min. The data were collected at 280 nm.

### Differential scanning calorimetry (DSC)

The DSC was performed on a MicroCal PEAQ-DSC system (Malvern Panalytical, Malvern, United Kingdom) with 0.5 mg/ml IgG under various conditions. Temperature scans were performed from 23°C to 100°C at a scan rate of 1°C/min. A buffer-buffer reference scan was subtracted from each sample scan prior to concentration normalization. All the data were analyzed using MicroCal PEAQ-DSC software.

### 8-anilino-1-naphthalene sulfonate (ANS) binding assay

The ANS was dissolved in DMSO to give a stock solution of 2.5 mM. Fluorescence measurements were performed at a concentration of 0.25 g/L mAb, and a 10-fold molar excess of ANS (relative to mAb) was used for fluorescence measurements. 100 μL of the mAb and ANS were thoroughly mixed and tested immediately. Fluorescence of ANS in the mAb solution was measured using a multimode micro-plate reader (Biotek Instruments Inc., Winooski, VT, United States) and 96-well clear membrane-bottomed microplates. Fluorescence emission was recorded at 490 nm after excitation at 403 nm. Measurements were taken every 0.75 min for 50 min.

### Spatial aggregation propensity (SAP) algorithm

The SAP algorithm was performed on the mAb1-IgG4 homology model using the BIOVIA Discovery Studio modeling environment, version 4.1 (Dassault Systèmes BIOVIA, San Diego, CA, United States). The homology model of mAb1-IgG4 was generated using the IgG4 template (Protein Data Bank code 5DK3).

### Screening of protein stabilizers

The mAb-IgG4 was dissolved in 25 mM citrate buffer (pH 3.5) supplemented with 50 mM amino acid (histidine, arginine, methionine, or glycine) or 10% w/v sugar (mannose or sucrose) to a final concentration of 1 mg/mL. The samples were then incubated under low pH conditions for 1 h. The percentage of protein aggregation was determined by analytical SEC, and thermodynamic parameters of mAb1-IgG4 in 25 mM citrate buffer with protein stabilizers were measured by DSC.

### Statistical analysis

All data were presented as the mean ± SEM. All statistical analyses were performed via GraphPad Prism 5 (GraphPad Software Inc., San Diego, CA, United States). The statistical analysis was compared by Student’s *t* test. Levels of *p < 0.05 were considered significant.

## Results

### Low pH-induced antibody aggregation

**FIGURE 1:**
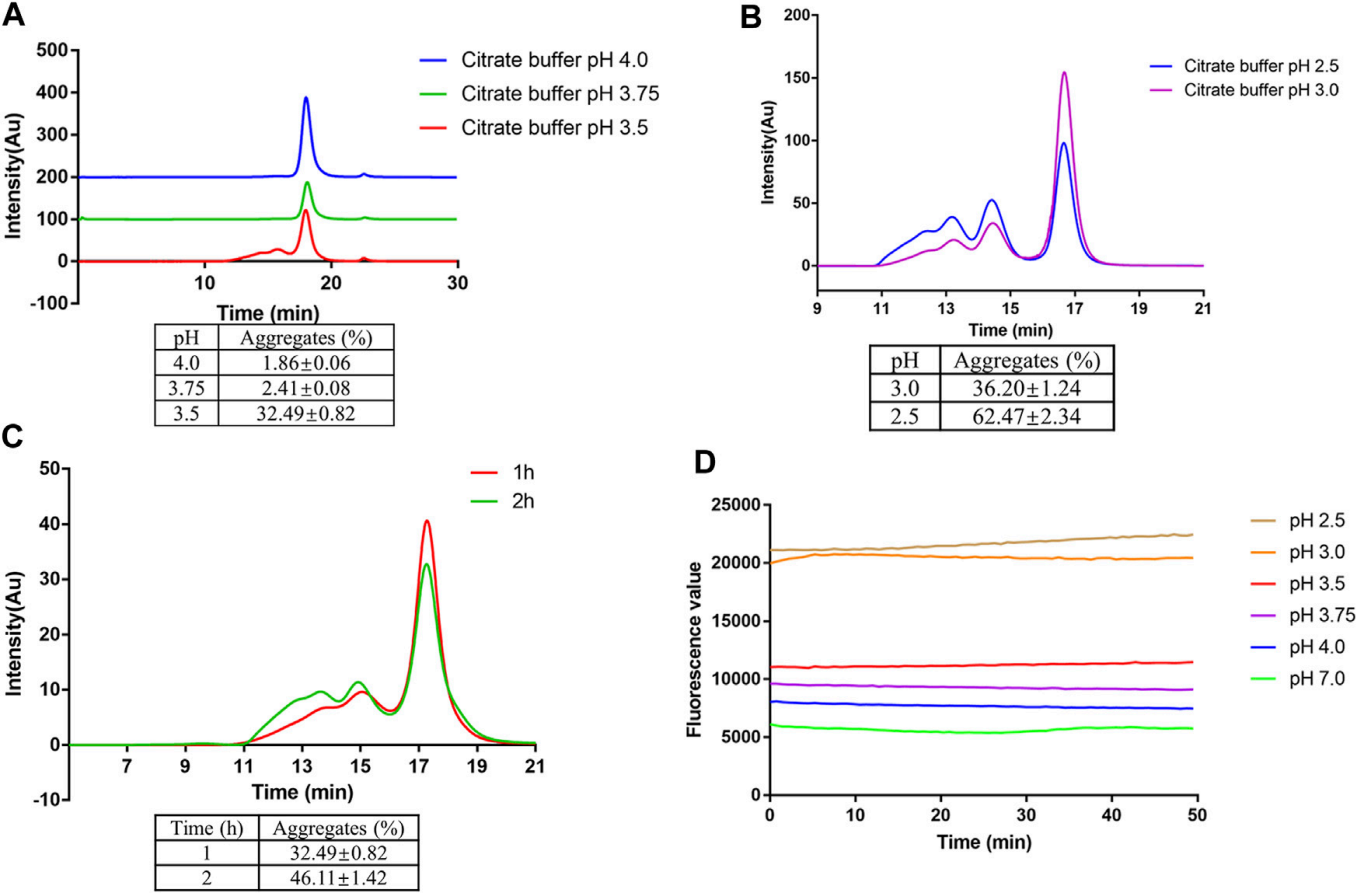
Low pH-induced aggregation of mAb1-IgG4. **(A)** mAb1-IgG4 was incubated in 25 mM citrate buffer at pH 3.5, 3.75 or 4.0 for 1 h at room temperature. The aggregation was analyzed by SEC-HPLC. **(B)** mAb1-IgG4 was incubated in 25 mM citrate buffer at pH 2.5 or 3.0 for 1 h at room temperature. The aggregation was analyzed by SEC-HPLC. **(C)** mAb1-IgG4 was incubated in 25 mM citrate buffer at pH 3.5 for 1 or 2 h at room temperature. The aggregation was analyzed by SEC-HPLC. **(D)** The exposure of hydrophobic patches on mAb1-IgG4 treated with 25 mM citrate buffer at pH 2.5, 3.0, 3.5, 3.75, 4.0 or 7.0 was analyzed by ANS binding assay. Data were presented as mean ± SEM. All experiments were repeated three times with consistent results.

To evaluate the stability of mAb1-IgG4 in a low pH solution, mAb1-IgG4 was incubated in 25 mM citrate buffer at pH 2.5 to 4.0 for 1 h at room temperature, followed by SEC-HPLC analysis. As shown in Figure 1A, mAb1-IgG4 was stable in 25 mM citrate buffer at pH 4.0 or 3.75; there was no significant protein aggregation, and the percentage of aggregates was less than 2.5%. However, mAb1-IgG4 aggregated significantly at pH 3.5; the percentage of aggregates increased to 32.49 ± 0.82%. When the pH was adjusted to 3.0 and 2.5, more aggregates were formed and the percentage of aggregates increased to 36.20 ± 1.24% and 62.47 ± 2.34%, respectively (Figure 1B). Further analysis revealed that the mAb1-IgG4 aggregation at low pH was time-dependent. As shown in Figure 1C, when mAb1-IgG4 was incubated in 25 mM citrate buffer at pH 3.5 for 1 or 2 h, the percentage of aggregates increased from 32.49 ± 0.82% to 46.11 ± 1.42%. These data indicated that most of the aggregates were formed during the first hour of incubation. The ANS binding assay was then performed to investigate the underlying mechanism of low pH-induced aggregation. The ANS anion is an extrinsic fluorescent probe and is conventionally considered to bind to pre-existing hydrophobic (non-polar) surfaces of proteins. Such binding is followed by an increase in ANS fluorescence intensity. As shown in Figure 1D, the fluorescence value, which reflects exposed hydrophobic surface areas, increased significantly with a decreasing pH gradient from 7.0 to 2.5. These results indicate that low pH-induced protein aggregation was most likely caused by conformational changes and the exposure of hydrophobic patches on the protein surface.

### The Fc domain determines the aggregation tendency of mAb1-IgG4

**FIGURE 2:**
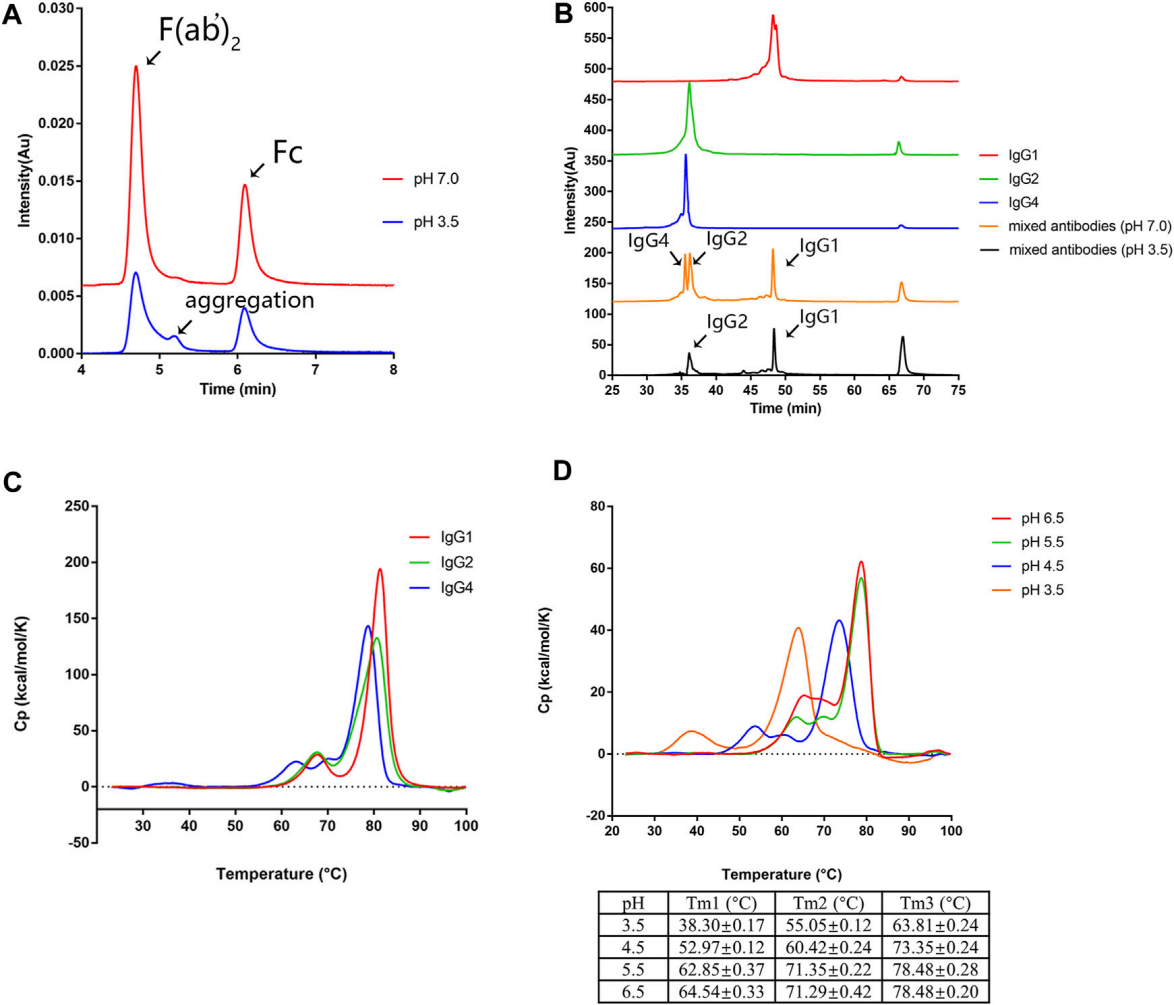
CH2 is the least stable domain under low pH conditions. **(A)** The mAb1-IgG4 was digested with the IdeS protease to obtain Fc and F (ab’)_2_ and incubated in 25 mM citrate buffer at pH 3.5 or pH 7.0 for 1 h. The aggregation was analyzed by SEC-UPLC. **(B)** mAb1-IgG1, mAb1-IgG2, and mAb1-IgG4 were incubated in 25 mM citrate buffer at pH 3.5 or pH 7.0 for 1 h. Individual and mixed mAb solution were analyzed by CEX-HPLC. **(C)** mAb1-IgG1, mAb1-IgG2, and mAb1-IgG4 diluted in 25 mM citrate buffer at pH 7.0 were analyzed by DSC. **(D)** mAb1-IgG4 diluted in 25 mM citrate buffer at pH 3.5, 4.5, 5.5, or 6.0 was analyzed by DSC. Data were presented as mean ± SEM. All experiments were repeated three times with consistent results.

It has been reported that variable domains (VH and VL), CH2 domain, and CH3 domain could initiate the aggregation process (Andersen et al., 2010; Brummitt et al., 2011; Dudgeon et al., 2012; Latypov et al., 2012; Iacob et al., 2013; Zhang-van Enk et al., 2013; Wu et al., 2014; Sakurai et al., 2015; Majumder et al., 2018; Namisaki et al., 2020). To further identify the potential aggregation-prone regions of mAb1-IgG4 under low pH conditions, mAb1-IgG4 was digested with IdeS protease to obtain F (ab’)2 and Fc. The antibody fragments were incubated in 25 mM citrate buffer (pH 3.5) or 25 mM citrate buffer (pH 7.4) for 1 h. Samples were then analyzed by SEC-UPLC. As shown in Figure 2A, compared to the sample incubated in citrate buffer (pH 7.4), there was a new peak (5.2 min) between F (ab’)2 (4.7 min) and Fc (6.1 min) in the sample incubated in low pH buffer. The new peak was identified by molecular weight as an Fc aggregate (5.2 min). This result indicated that the Fc domain determines the aggregation tendency of mAb1-IgG4.

To support this conclusion, the variable domains (VH and VL) of mAb1-IgG4 were grafted onto the constant regions of IgG1 and IgG2 to generate mAb1-IgG1 and mAb1-IgG2. CEX has been used as an orthogonal method to analyze the aggregation of mAbs based on the loss of soluble monomers in individual or mixed antibody solutions (Chen et al., 2010; Hari et al., 2010). mAb1-IgG1, mAb1-IgG2, and mAb1-IgG4 were incubated in 25 mM citrate buffer at pH 3.5 or pH 7.0 for 1 h. Samples were then analyzed by CEX. As shown in Figure 2B, mAb1-IgG1 has a longer retention time than that of mAb1-IgG2 and mAb1-Ig4, which could be attributed to the relatively more basic amino acids in IgG1. mAb1-Ig2 and mAb1-IgG4 exhibited significant reductions in monomers and increases in aggregates after low pH treatment. However, mAb1-IgG4 was the least stable IgG subclass under low pH conditions. The order of the IgG subclass stability under acidic conditions was IgG1>IgG2>IgG4. These data are consistent with previous reports that the IgG4 subclass is more prone to aggregation under low pH conditions (Skamris et al., 2016). Taken together, the Fc domain determines the aggregation of mAb1-IgG4.

### CH2 is the least stable domain at low pH

To further determine which part of the mAb1-IgG4 Fc domain contributes to aggregation formation under low pH conditions, the thermal stability of mAb1-IgG1, mAb1-IgG2, and mAb1-IgG4 was analyzed by DSC. As shown in Figure 2C and Table 1, the curve of mAb1-IgG4 shows three transitions: one with a denaturation temperature of Tm1 at 62.25 ± 0.27°C, a second (Tm2) at 68.68 ± 0.20°C, and a third (Tm3) at 78.67 ± 0.21°C. Tm1, Tm2, and Tm3 represent the denaturation temperatures for CH2, CH3, and Fab, respectively. The curves of mAb1-IgG1 and mAb1-IgG2 showed two transitions: Tm1 at 67.12 ± 0.10°C and 66.94 ± 0.21°C, and Tm2 at 81.13 ± 0.04°C and 80.49 ± 0.31°C, respectively. For mAb1-IgG1 and mAb1-IgG2, Tm1 and Tm2 represent the denaturation temperatures for CH2 and Fab/CH3. These data indicate that the CH2 domain of IgG4 is less stable than that of IgG1 and IgG2. In addition, the CH3 and Fab of IgG4 are relatively less stable than those of IgG1 and IgG2. For all three subclasses of mAb1, the conformational denaturation is initiated at CH2 under thermal stress.

**TABLE 1:** Thermal stability analysis of mAb1-IgG1, mAb1-IgG2, and mAb1-IgG4 by DSC.

Subclass	Tm1 (°C)	Tm2 (°C)	Tm3 (°C)
mAb1-IgG1	67.12 ± 0.10 (CH2)	81.13 ± 0.04 (Fab/CH3)	
mAb1-IgG2	66.94 ± 0.21 (CH2)	80.49 ± 0.31 (Fab/CH3)	
mAb1-IgG4	62.25 ± 0.27 (CH2)	68.68 ± 0.20 (CH3)	78.67 ± 0.21 (Fab)

We then investigated the effect of low pH on the thermal stability of mAb1-IgG4. mAb1-IgG4 diluted in 25 mM citrate buffer at pH 3.5, 4.5, 5.5, and 6.5 was analyzed by DSC. The temperature-induced unfolding of the antibodies at different pH is shown in Figure 2D. The Tm1, Tm2, and Tm3 of mAb1-IgG4 were 64.54 ± 0.33°C, 71.29 ± 0.42°C, and 78.48 ± 0.20°C at pH 6.5, and 38.30 ± 0.17°C, 55.05 ± 0.12°C, and 63.81 ± 0.24°C at pH 3.5. The ΔTm1 (Tm1 at pH 6.5-Tm1 at pH 3.5), ΔTm2 (Tm2 at pH 6.5-Tm2 at pH 3.5), and ΔTm3 (Tm3 at pH 6.5-Tm3 at pH 3.5) were 26.24°C, 16.24°C, and 14.67°C, respectively. It is evident that acidic conditions have a more significant effect on Tm1 than on Tm2 and Tm3. We proposed that the stability of the CH2 domain is the predominant factor that contributes to low pH-induced aggregation, and engineering of CH2 may alleviate low pH-induced aggregation.

### L309E, Q311D, and Q311E mutants reduce low pH-induced aggregation

**FIGURE 3:**
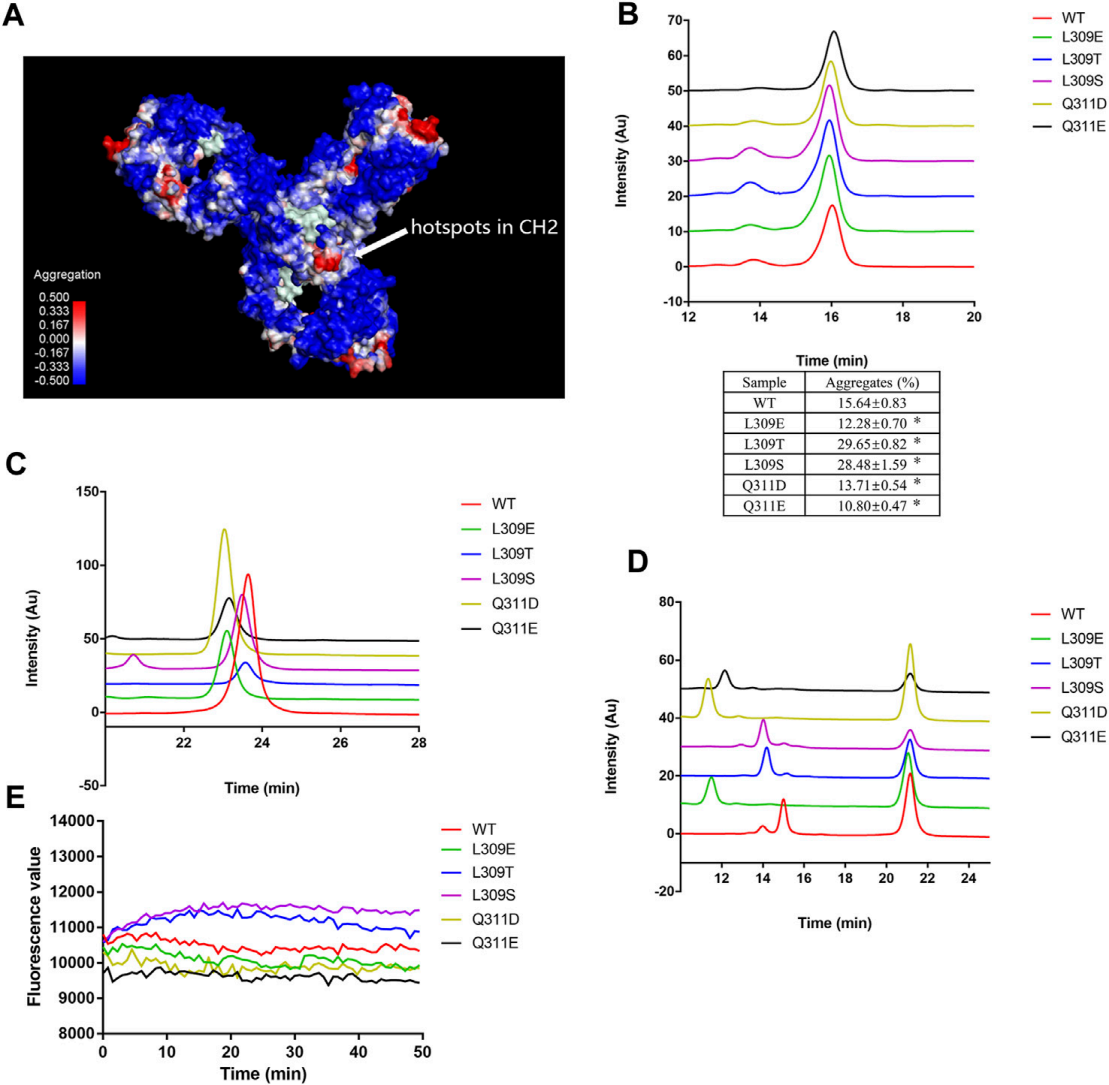
L309E, Q311D, and Q311E mutants reduced low pH-induced aggregation. **(A)** The aggregation hotspots on mAb1-IgG4 were predicted with the SAP software. The arrow indicates the hotspots on the CH2 domain, which includes Leu 251, Met 252, Ile 253, Ser 254, Arg 255, Thr 256, Thr 307, Leu 309, and Gln311. **(B)** The mutants and wild type mAb1-IgG4 were incubated in 25 mM citrate buffer at pH 3.5 for 1 h and analyzed by SEC-HPLC. **(C)** The mutants and wild type mAb1-IgG4 were incubated in 25 mM citrate buffer at pH 3.5 for 1 h and analyzed by HIC-HPLC. **(D)** The mutants and wild type mAb1-IgG4 were digested with IdeS and analyzed by HIC-HPLC. **(E)** The mutants and wild type mAb1-IgG4 were incubated in 25 mM citrate buffer at pH 3.5 for 1 h. The exposure of hydrophobic patches was analyzed by ANS binding assay. Data were presented as mean ± SEM. *p < 0.05, significantly different from WT. All experiments were repeated three times with consistent results.

Hydrophobicity interaction is the most important non-covalent force in protein aggregation formation (Münch and Bertolotti, 2010). The SAP algorithm incorporates structural and sequence information to identify motifs that contribute to protein aggregation (Clark et al., 2014). We used the SAP algorithm to estimate the aggregation motifs of mAb1-IgG4. The SAP algorithm predicts that Leu 251, Met 252, Ile 253, Ser 254, Arg 255, Thr 256, Thr 307, Leu 309, and Gln311 on CH2 are hotspots for Fc-Fc self-association (Figure 3A). The Fc domain, particularly the CH2 domain, showed high SAP values, indicating their higher tendency to self-association. Residues Ile253, Phe296 and Leu309 were reported to be responsible for the IgG4 Fc-Fc packing interaction (Davies et al., 2014).

Based on SAP calculations and IgG4 Fc-Fc packing interfaces, we selected L309 and Q311 for mutation analysis. We proposed that mutating leucine or glutamine to other more polar and hydrophilic amino acids may attenuate the hydrophobic interaction between IgG4 Fc and Fc. L309E, L309T, L309S, Q311D, and Q311E mutations were introduced into mAb1-IgG4. All the mutants and wild-type mAb1-IgG4 were incubated in 25 mM citrate buffer (pH 3.5) for 1 h and analyzed by SEC-HPLC. As shown in Figure 3B, mAb1-IgG4-L309E, mAb1-IgG4-Q311D, and mAb1-IgG4-Q311E mutants formed fewer aggregates under low pH conditions compared to wild-type mAb1-IgG4 (WT), whereas L309T and L309S mutants formed more aggregates under low pH conditions. To explore the possible mechanism of point mutation induced aggregation propensities, all the mutants and wild type mAb1-IgG4 were analyzed by HIC. Compared to wild type mAb1-IgG4, mAb1-IgG4-L309E, mAb1-IgG4-Q311D, and mAb1-IgG4-Q311E mutants were eluted earlier, indicating decreased hydrophobicity (Figure 3C). To further investigate the hydrophobicity of the Fc domain, mAb1-IgG4 (WT) and mAb1-IgG4 mutants were digested with IdeS protease and analyzed by HIC. Compared to wild type mAb1-IgG4 Fc, all the mutants show decreased hydrophobicity at different levels. However, mAb1-IgG4-L309E, mAb1-IgG4-Q311D, and mAb1-IgG4-Q311E showed a greater reduction in hydrophobicity than the others (Figure 3D). However, these data were unable to fully explain the heterogeneity in aggregation propensity, as the L309T and L309S mutants formed more aggregates than the wild type under low pH treatment. It was possible that an increase in overall hydrophilicity did not always turn into reduced hydrophobic patches or aggregation-prone regions. The exposure of hydrophobic patches on wild-type mAb1-IgG4 and mutants was analyzed by the ANS binding assay. As shown in Figure 3E, L309E, Q311D, and Q311E mutants showed lower fluorescence levels compared to L309T, L309S, and wild-type mAb1-IgG4. These data may explain why only these three mutants effectively attenuate the aggregation tendency of mAb1-IgG4. Taken together, the L309E, Q311D, and Q311E mutants partially attenuated mAb1-IgG4 aggregation under low pH conditions, most likely by reducing the hydrophobicity of the CH2 domain.

### Sucrose and mannose can attenuate low pH-induced mAb1-IgG4 aggregation

**FIGURE 4:**
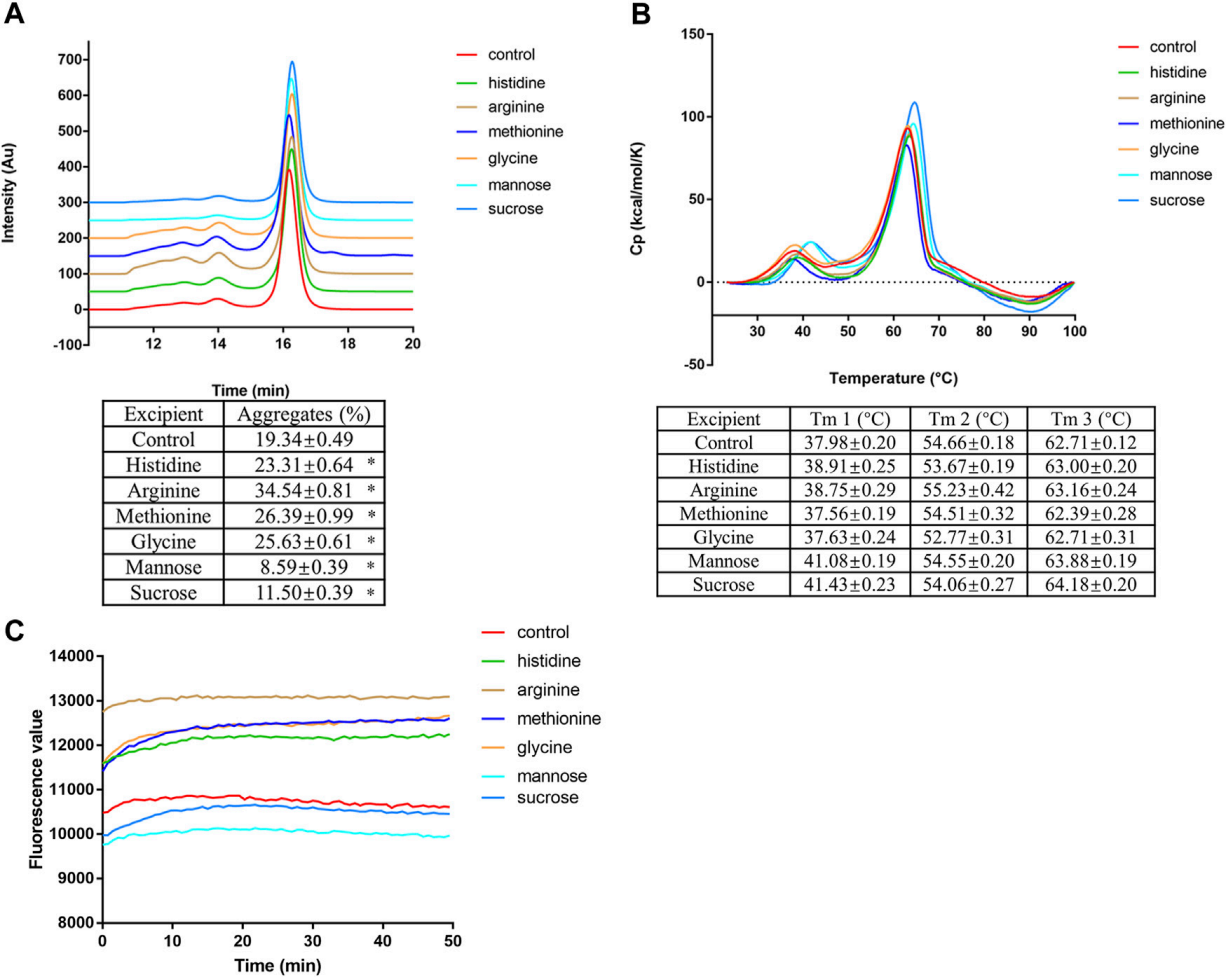
Sucrose and mannose can attenuate low pH-induced mAb1-IgG4 aggregation. **(A)** mAb1-IgG4 was incubated in 25 mM citrate buffer (pH 3.5) supplemented with histidine, arginine, methionine, glycine, mannose or sucrose for 1 h and analyzed by SEC-HPLC. **(B)** mAb1-IgG4 diluted in 25 mM citrate buffer (pH 3.5) supplemented with histidine, arginine, methionine, glycine, mannose or sucrose were analyzed by DSC. **(C)** mAb1-IgG4 was incubated in 25 mM citrate buffer (pH 3.5) supplemented with histidine, arginine, methionine, glycine, mannose or sucrose. The exposure of hydrophobic patches was analyzed by ANS binding assay. Data were presented as mean ± SEM. *p < 0.05, significantly different from control. All experiments were repeated three times with consistent results.

Since amino acid point mutations that decrease the hydrophobicity of the Fc domain could partially attenuate protein aggregation under low pH conditions, we proposed that protein stabilizers that could shield hydrophobic patches on proteins would be effective in preventing mAb1-IgG4 aggregation under low pH conditions. mAb1-IgG4 was incubated in 25 mM citrate buffer (pH 3.5) supplemented with histidine, arginine, methionine, glycine, mannose, or sucrose for 1 h and analyzed by SEC-HPLC. As shown in Figure 4A, the percentage of aggregates in the control (no supplementation) is 19.34 ± 0.49%; the addition of histidine, arginine, methionine, and glycine did not attenuate the aggregation but exacerbated the aggregation, the percentage of aggregates was 23.31 ± 0.64%, 34.54 ± 0.81%, 26.39 ± 0.99%, and 25.63 ± 0.61%, respectively. However, supplementing with mannose and sucrose significantly decreased the aggregation of antibodies; the percentage of aggregates was 8.59 ± 0.39% and 11.50 ± 0.39% respectively.

DSC was conducted to detect the effect of amino acids and sugars on the thermal stability of mAb1-IgG4 in a low pH solution. As shown in Figure 4B, amino acid supplementation had no effect on the melting temperature compared to the control. Supplementation with mannose and sucrose increased the Tm1, the denaturation temperature of the CH2 domain; the ΔTm1 is 3.1°C and 3.45°C, respectively. We then have semi-quantified the exposed hydrophobic patches on mAb1-IgG4 with the ANS binding assay. Compared to the control and amino acids supplementation, the addition of mannose and sucrose showed a lower fluorescence value, indicating lower ANS binding and fewer exposed hydrophobic patches (Figure 4C). Taken together, the results showed that sucrose and mannose were effective in attenuating the mAb1-IgG4 aggregation under low pH conditions, probably by shielding hydrophobic patches and increasing the thermal stability of the CH2 domain. These stabilizers are pharmacologically inert, and could be easily removed in further downstream procedures.

## Discussion

IgG4 is suitable for use in either antagonist or agonist format therapeutic antibodies because it is largely unable to activate antibody-dependent immune effector responses. During the development of an IgG4 antibody, mAb1-IgG4, we have encountered protein aggregation during protein A purification and integral low pH viral inactivation in 25 mM citrate buffer at pH 3.5. The percentage of aggregate area increased with decreasing pH. Protein A affinity chromatography is commonly applied in the purification of antibody products due to its high selectivity and effective removal of impurities (Shukla et al., 2007). Mammalian cell expression systems intrinsically pose a risk of viral contamination. The ICH Q5A requires viral reduction procedures to assure the safety of products. Temporary exposure to low pH is known to be a robust procedure for inactivating enveloped viruses. Since the elution of mAbs from the protein A column is achieved by reducing the mobile phase pH to a range of 3–4 (Pabst et al., 2018), it is relatively easy to incorporate a low pH incubation step (Durno and Tounekti, 2015). However, exposure of mAbs under low pH conditions can cause protein aggregation and loss of yield (Skamris et al., 2016; Wälchli et al., 2020).

IgG is a multi-domain protein consisting of variable and constant domains; it has been reported that the CH2, CH3, or Fab domain can initiate protein aggregation (Andersen et al., 2010; Brummitt et al., 2011; Dudgeon et al., 2012; Latypov et al., 2012; Iacob et al., 2013; Zhang-van Enk et al., 2013; Wu et al., 2014; Sakurai et al., 2015; Majumder et al., 2018; Namisaki et al., 2020). For mAb1-IgG4, we used several analytical methods to figure out that stability of the CH2 domain is the main determining factor in aggregate formation. The SEC-UPLC analysis shows that only the Fc domain aggregates under low pH conditions, although the Fc domain is less hydrophobic than F (ab’)2 as revealed by HIC-HPLC. Since Fc is composed of CH2 and CH3 domains, the DSC was conducted to confirm which part is the primary contributor to the aggregation. DSC data indicate that the CH2 domain is the least stable of all IgG domains under neutral and low pH conditions. Additionally, DSC data show that the CH2 domain has a significantly higher ΔTm1(Tm1 at pH 6.5 - Tm1 at pH 3.5) than the CH3 domain, indicating that CH2 is more sensitive to low pH conditions.

Amino acid sequences, or the intrinsic properties of antibodies, determine their conformational structure, surface charge distribution, hydrophobicity, and tendency to aggregate. A human antibody is composed of 12 immunoglobulin domains, which are designated as VL, CL, VH, CH1, CH2, and CH3. A particular immunoglobulin fold is composed of 70–110 amino acids, which form a beta-sandwich of seven or more strands in two sheets (Wang, 2013). The CH2 domain is an important and unique component of human IgG. It has unique properties, 1), CH2 connects with the lower hinge region, and they are relatively hydrophobic and structurally flexible, which is necessary for interaction with Fcγ receptors (Sibéril et al., 2006; Kiyoshi et al., 2015); 2), the CH2-CH2 inter-chain interaction is mediated by N-glycans, not by direct protein interaction (Kayser et al., 2011; Estes et al., 2021); 3), CH2 and CH3 interact with FcRn, which is mediated by electrostatic forces and hydrophobic interaction; 4), CH2 is glycosylated at N297, which partially but not completely covers hydrophobic patches on CH2 (Kayser et al., 2011; Kiyoshi et al., 2017). N-glycosylation contributes to antibody stability and the tendency to aggregation. The absence of N-linked oligosaccharides or incomplete N-glycosylation promotes the formation of antibody aggregation (Mimura et al., 2000; Krapp et al., 2003; Kayser et al., 2011; Onitsuka et al., 2014; Li et al., 2021). In short, CH2 is a hetero-glycosylated immunoglobulin domain that is structurally less rigid, more flexible, more hydrophobic, and more sensitive to unfolding and aggregation induced by external stresses.

Aggregation propensity is directly related with protein surface hydrophobicity (Galm et al., 2017). We propose that reducing the hydrophobicity of CH2 may attenuate antibody aggregation under low pH conditions. L309 and Q311 were critical amino acids involved in IgG4 Fc-Fc packing and aggregate formation (Davies et al., 2014). Mutating L309 and Q311 to more polar and hydrophilic amino acids could theoretically reduce protein hydrophobicity and attenuate mAb1-IgG4 aggregation under low pH conditions. Mutants Q311D, Q311E, and L309E, which greatly reduced Fc hydrophobicity, partially attenuated the mAb1-IgG4 aggregation under low pH conditions. A previous study reported that the Q295F/Y296A mutant conferred enhanced CH2 stability against thermal and low pH-induced aggregation in the context of an IgG1 antibody (Chen et al., 2016). The underlying mechanism of the Q295F/Y296A mutation is the introduction of a non-covalent interaction between aromatic amino acid, F295, and the N-glycan, which is different from the tactic used in this study.

Consistent with our findings, previous studies have demonstrated that sugars can interact with aromatic residues on the antibody surface, which can help prevent hydrophobic interactions (Cloutier et al., 2019). In our study, mannose and sucrose increased the Tm of the CH2 domain and substantially decreased low pH-induced aggregation. The underlying mechanism should be the shielding of hydrophobic patches on mAb1-IgG4, as revealed by the ANS binding assay.

From a thermodynamic perspective, protein aggregation could be considered as a temperature sensitive, entropy-driven, and spontaneously occurring process that is characterized by partial or complete collapse of the conformational structure, exposure of hydrophobic side chains, and assembly of monomers (Shalaby and Lauffer, 1985; Licht et al., 1999; Morris et al., 2009; Li et al., 2016). In most cases, non-covalent interactions dominate the transformation from ordered structures to disordered oligomers. The hydrophobic interaction, which is entropy-driven and intrinsically temperature sensitive, plays a key role in protein aggregation (Bennion and Daggett, 2003). The aggregation of many proteins has been shown to exhibit a positive enthalpy and entropy. The positive enthalpy indicates that protein aggregation is an endothermic reaction, and the rate of aggregation is positively correlated with the surrounding temperature (Licht et al., 1999; Morris et al., 2009). The high positive entropy is believed to result from the release of water molecules upon aggregation. We cannot completely counteract the law of entropy increase and an entropy-driven process, the only feasible strategy is to slow it down. In this study, we successfully slowed down this process by introducing more polar and hydrophilic amino acids into the CH2 domain and by adding protein stabilizers, mannose and sucrose, to cover the hydrophobic patches. Our findings provide new information on the stability of IgG4 subclass antibodies under acidic conditions and offer new perspectives for the design and engineering of therapeutic antibodies with enhanced stability.

## Data Availability

The original contributions presented in the study are included in the article/supplementary material, further inquiries can be directed to the corresponding authors.
